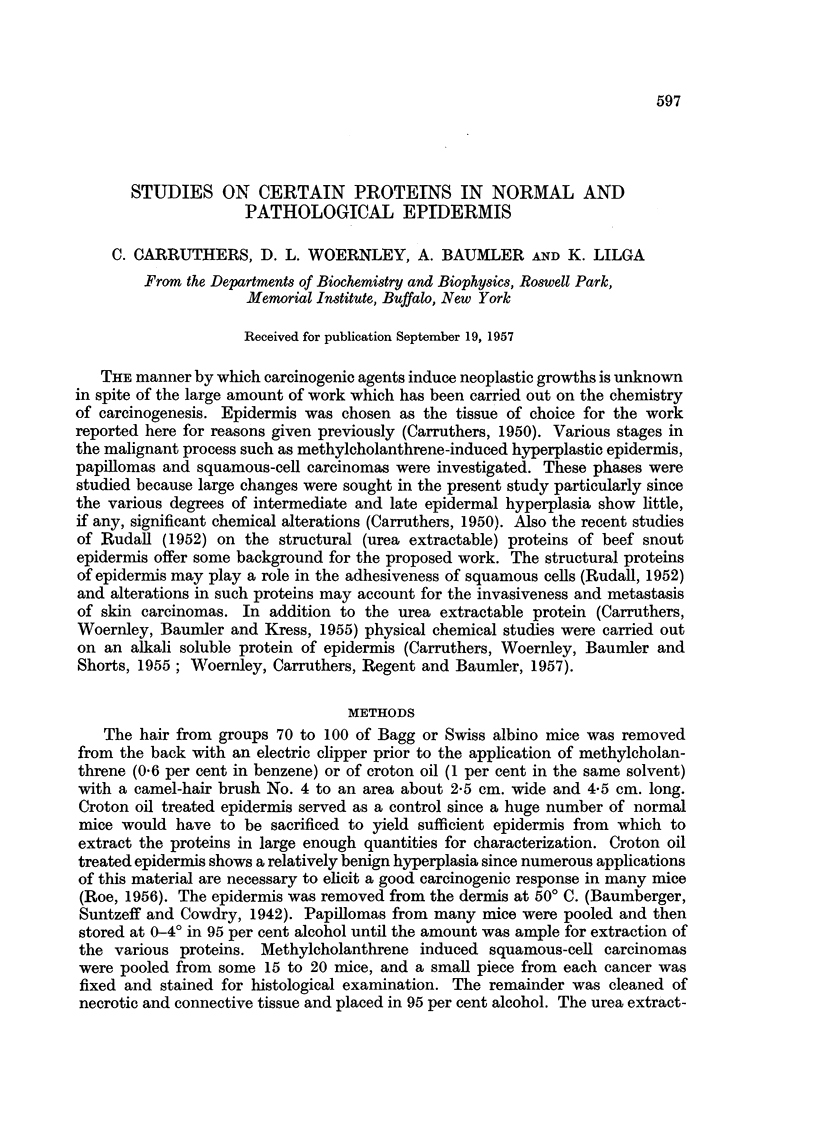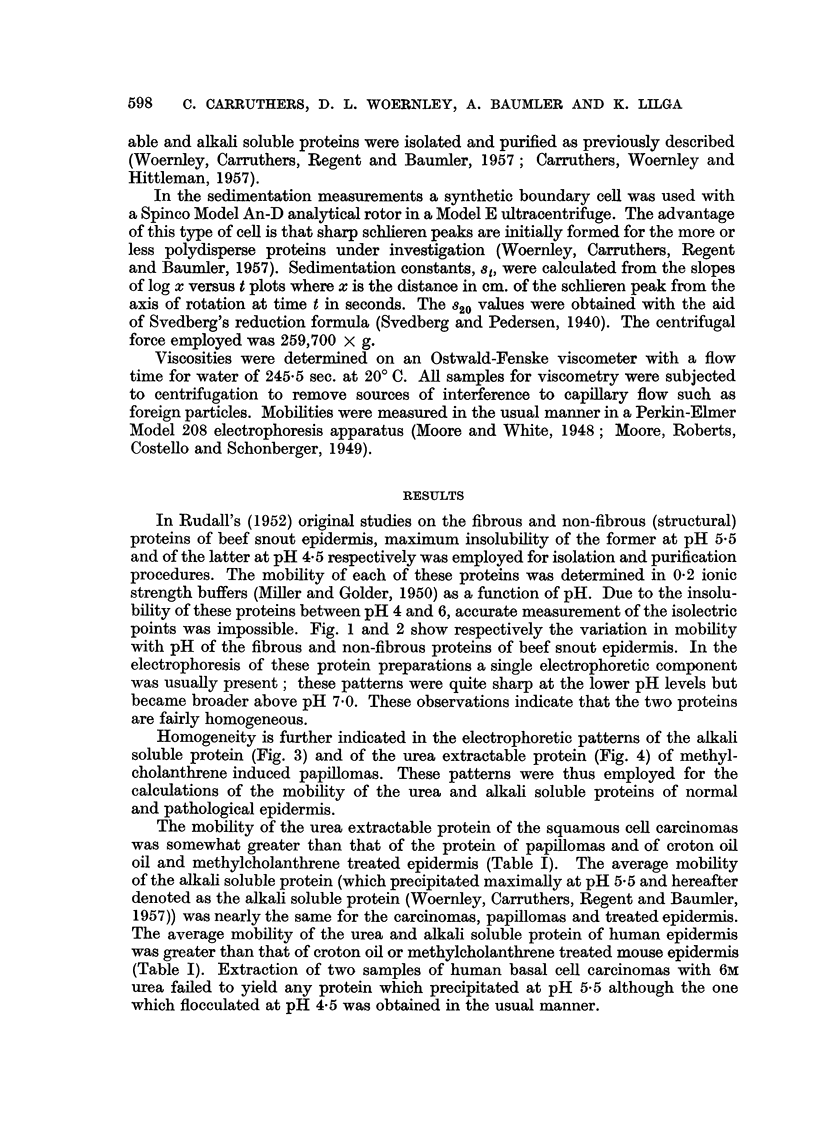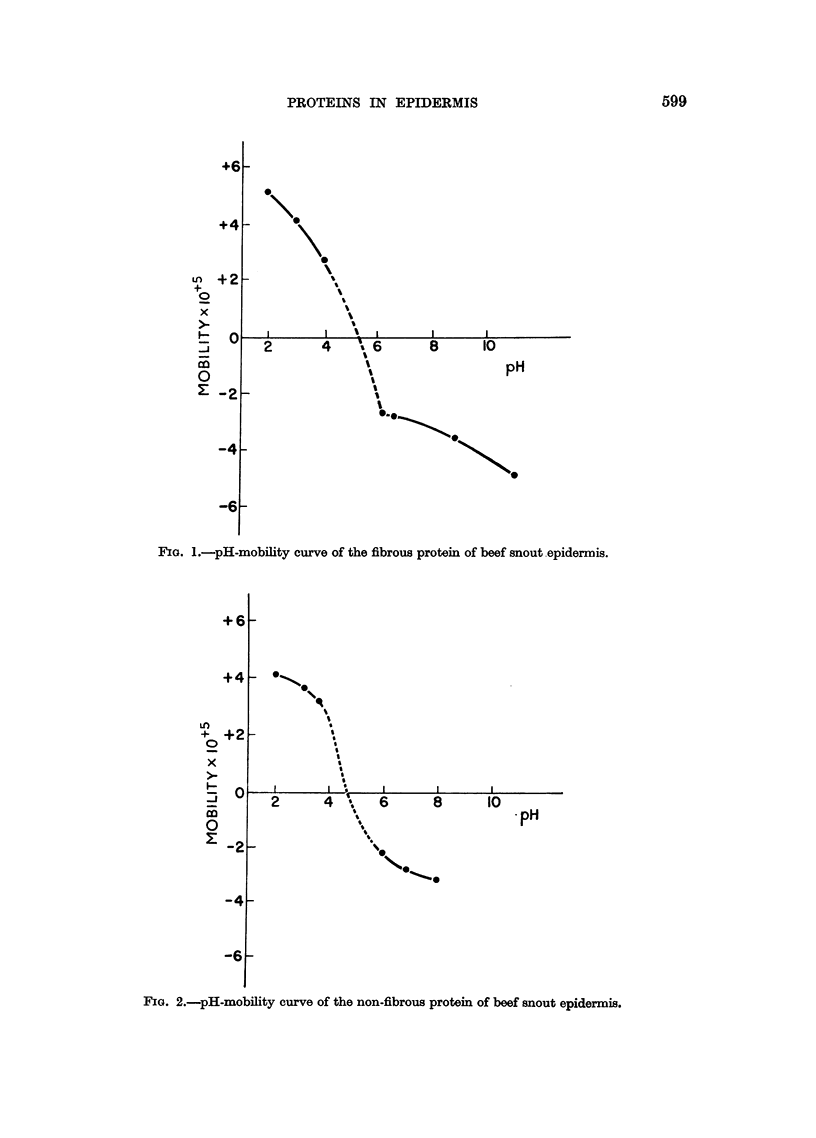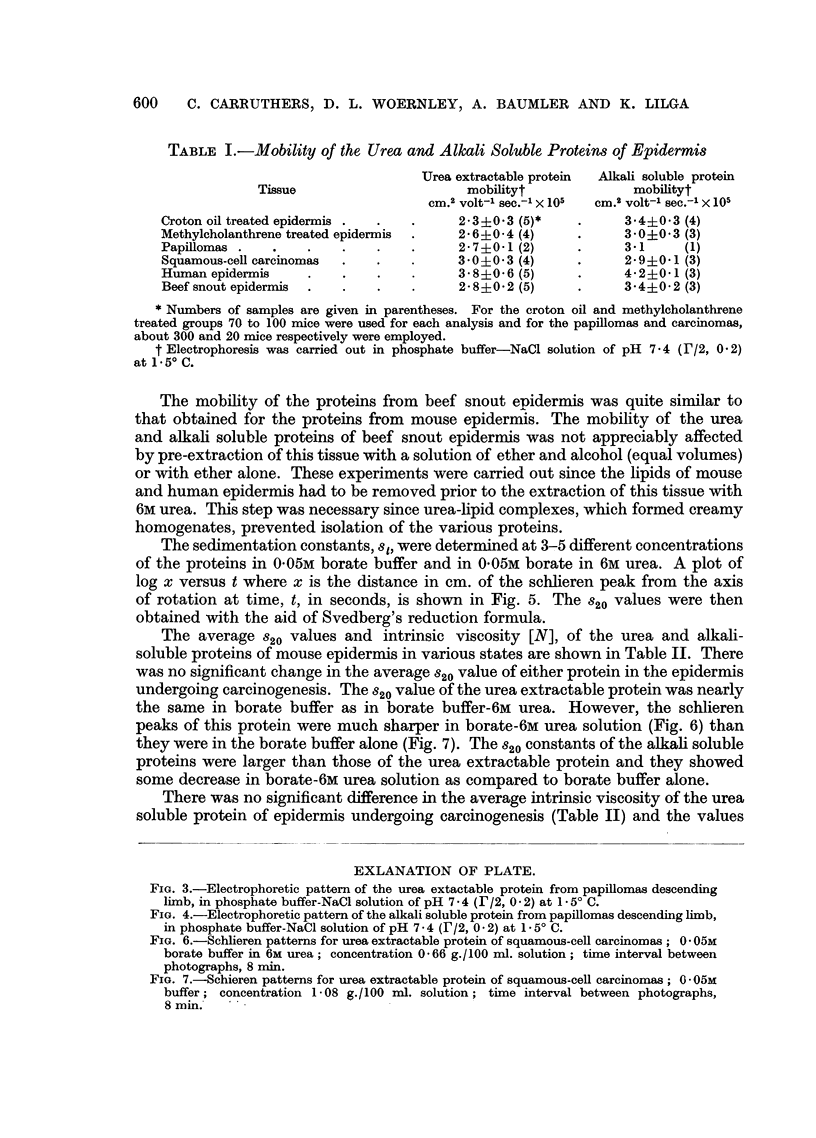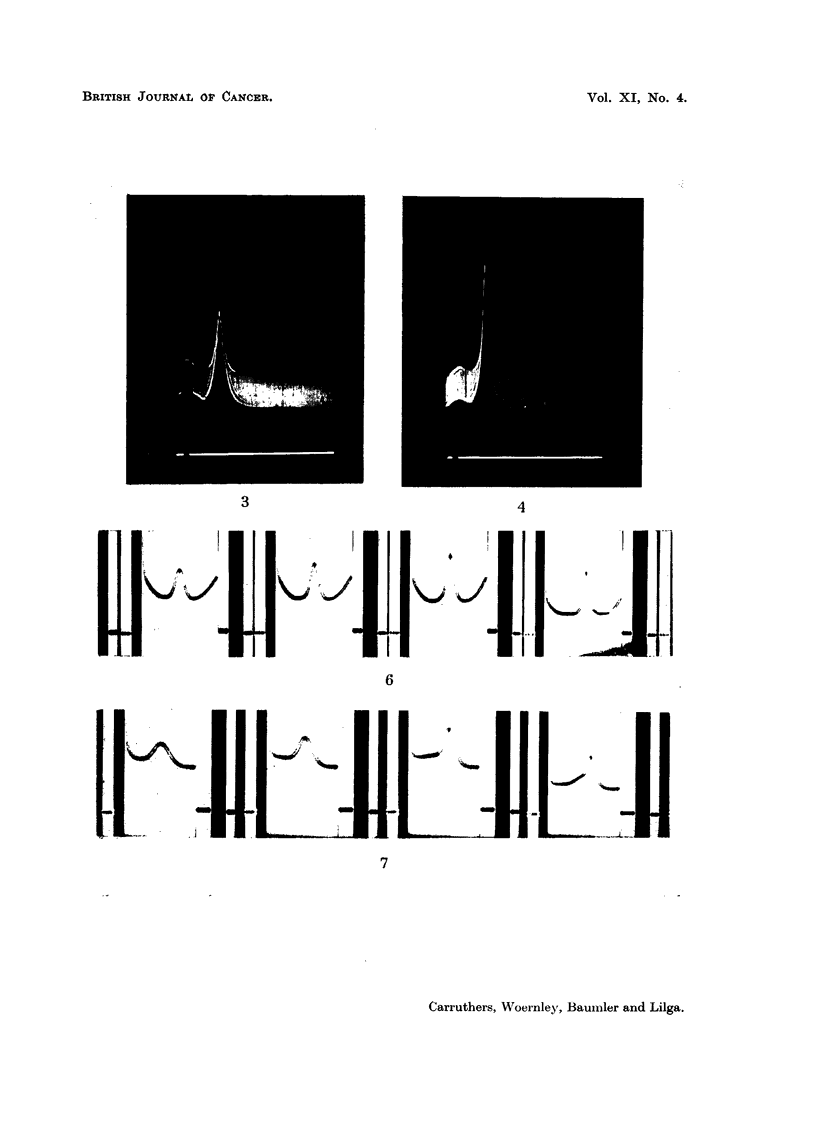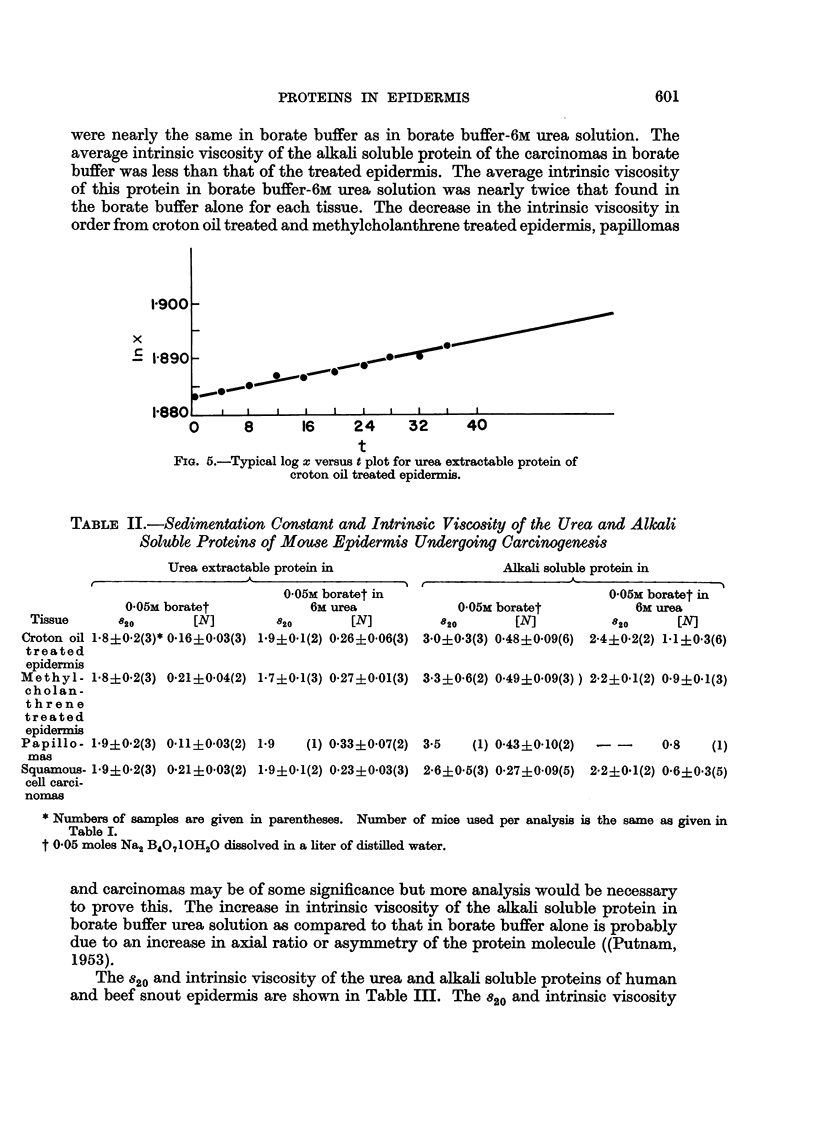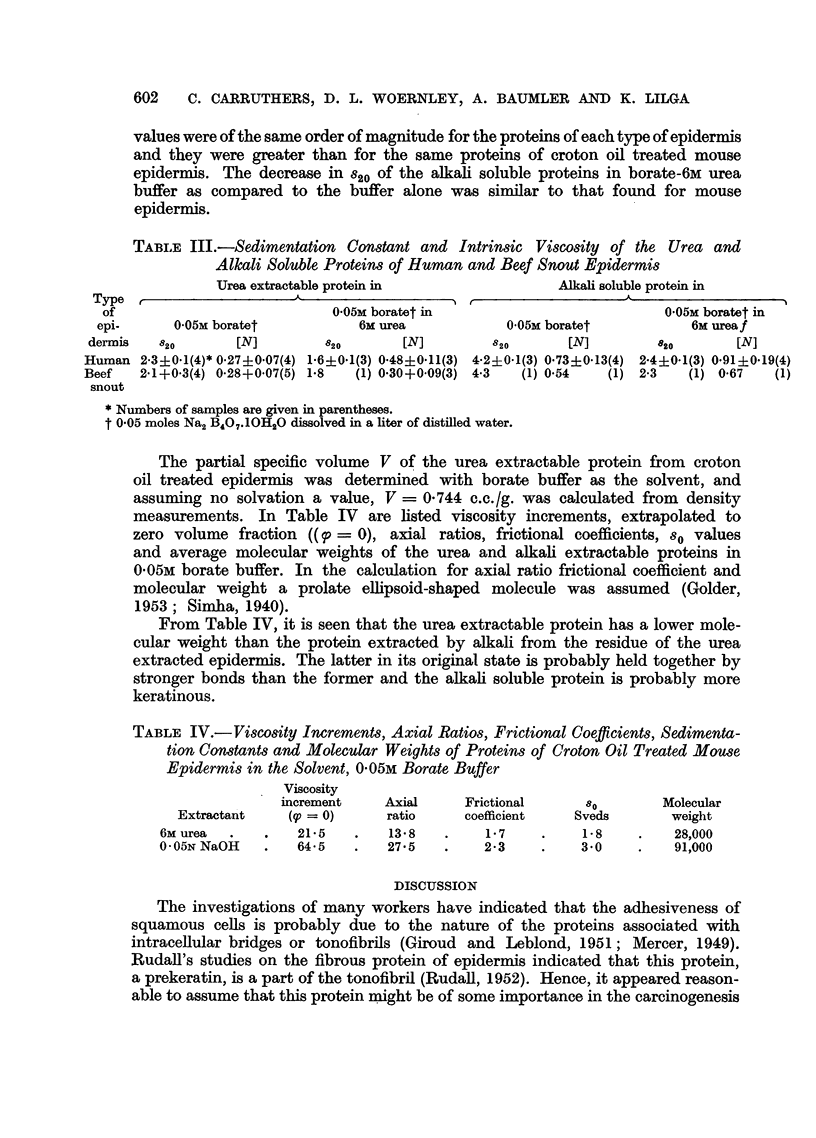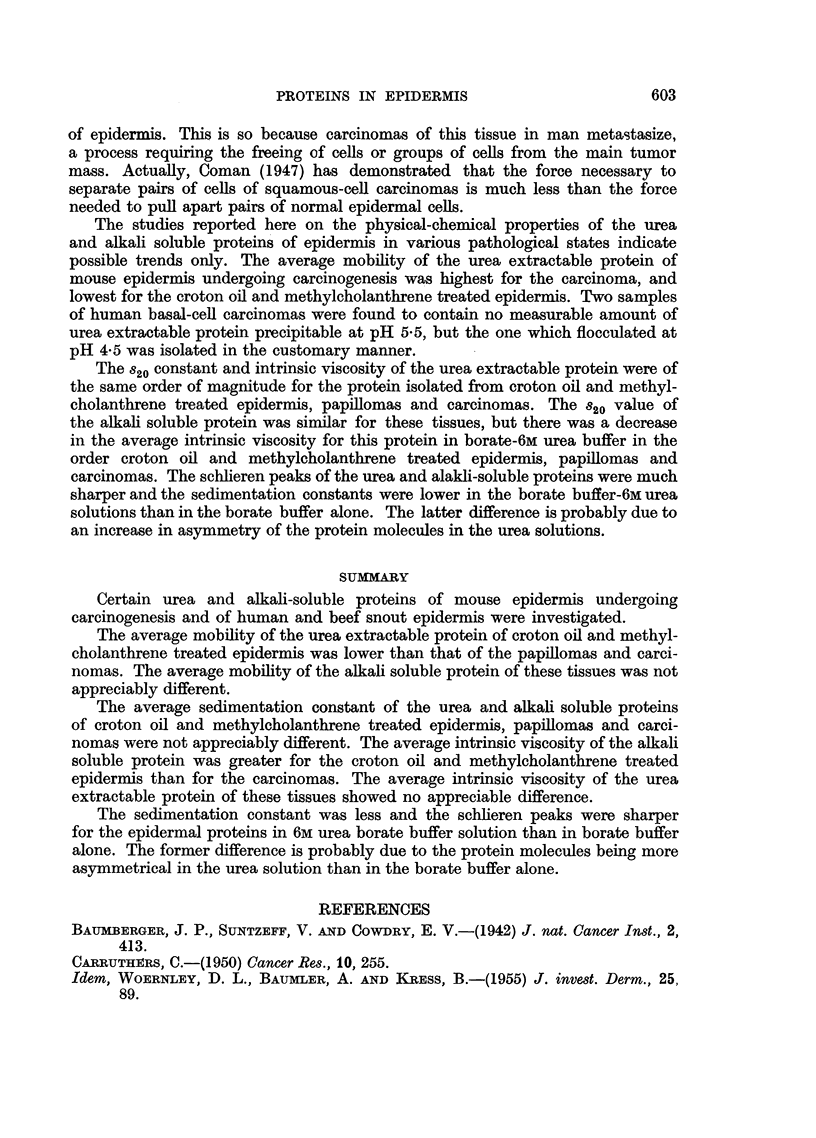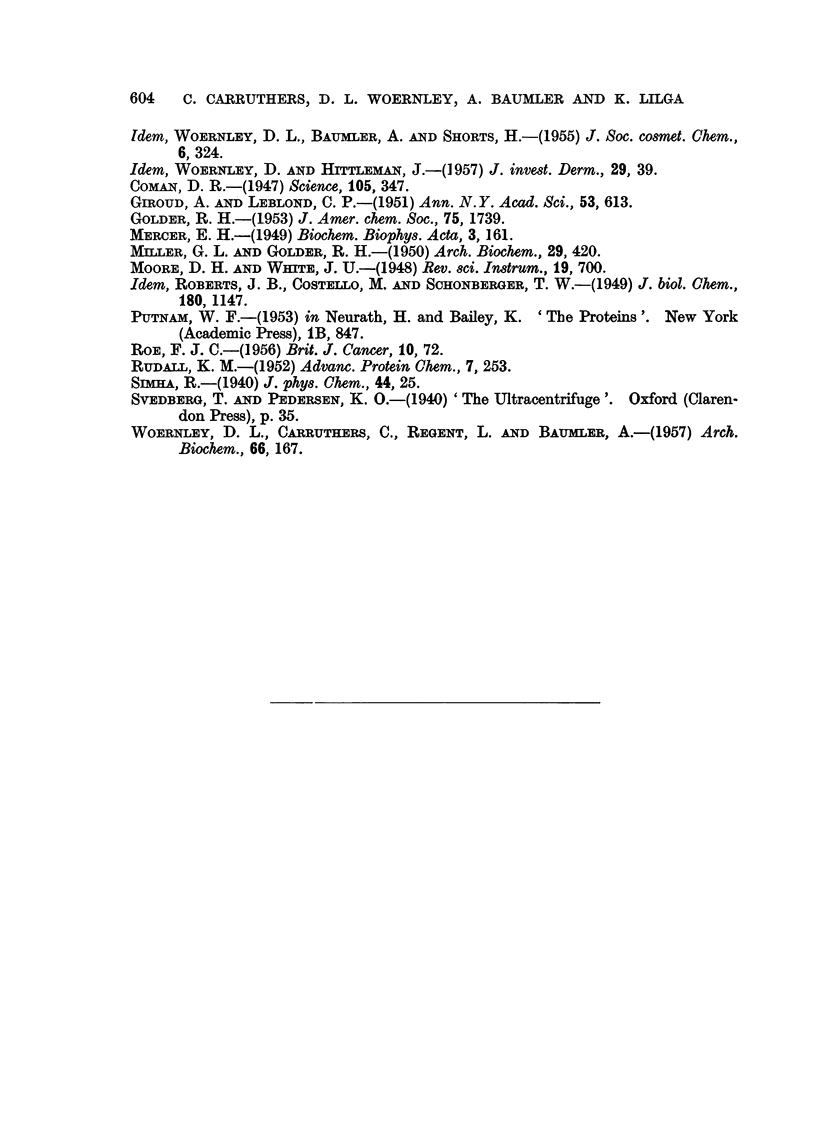# Studies on Certain Proteins in Normal and Pathological Epidermis

**DOI:** 10.1038/bjc.1957.73

**Published:** 1957-12

**Authors:** C. Carruthers, D. L. Woernley, A. Baumler, K. Lilga

## Abstract

**Images:**


### STUDIES ON CERTAIN PROTEINS IN NORMAL AND PATHOLOGICAL EPIDERMIS

C. CARRUTHERS, D. L. WOERNLEY, A. BAUMLER AND K. LILGA

*From the Departments of Biochemistry and Biophysics, Roswell Park, Memorial Institute, Buffalo, New York*

Received for publication September 19, 1957

THE manner by which carcinogenic agents induce neoplastic growths is unknown in spite of the large amount of work which has been carried out on the chemistry of carcinogenesis. Epidermis was chosen as the tissue of choice for the work reported here for reasons given previously (Carruthers, 1950). Various stages in the malignant process such as methylcholanthrene-induced hyperplastic epidermis, papillomas and squamous-cell carcinomas were investigated. These phases were studied because large changes were sought in the present study particularly since the various degrees of intermediate and late epidermal hyperplasia show little, if any, significant chemical alterations (Carruthers, 1950). Also the recent studies of Rudall (1952) on the structural (urea extractable) proteins of beef snout epidermis offer some background for the proposed work. The structural proteins of epidermis may play a role in the adhesiveness of squamous cells (Rudall, 1952) and alterations in such proteins may account for the invasiveness and metastasis of skin carcinomas. In addition to the urea extractable protein (Carruthers, Woernley, Baumler and Kress, 1955) physical chemical studies were carried out on an alkali soluble protein of epidermis (Carruthers, Woernley, Baumler and Shorts, 1955 ; Woernley, Carruthers, Regent and Baumler, 1957).

### METHODS

The hair from groups 70 to 100 of Bagg or Swiss albino mice was removed from the back with an electric clipper prior to the application of methylcholanthrene (0·6 per cent in benzene) or of croton oil (1 per cent in the same solvent) with a camel-hair brush No. 4 to an area about 2·5 cm. wide and 4·5 cm. long. Croton oil treated epidermis served as a control since a huge number of normal mice would have to be sacrificed to yield sufficient epidermis from which to extract the proteins in large enough quantities for characterization. Croton oil treated epidermis shows a relatively benign hyperplasia since numerous applications of this material are necessary to elicit a good carcinogenic response in many mice (Roe, 1956). The epidermis was removed from the dermis at 50° C. (Baumberger, Suntzeff and Cowdry, 1942). Papillomas from many mice were pooled and then stored at 0–4° in 95 per cent alcohol until the amount was ample for extraction of the various proteins. Methylcholanthrene induced squamous-cell carcinomas were pooled from some 15 to 20 mice, and a small piece from each cancer was fixed and stained for histological examination. The remainder was cleaned of necrotic and connective tissue and placed in 95 per cent alcohol. The urea extractable and alkali soluble proteins were isolated and purified as previously described (Woernley, Carruthers, Regent and Baumler, 1957; Carruthers, Woernley and Hittleman, 1957).

In the sedimentation measurements a synthetic boundary cell was used with a Spinco Model An-D analytical rotor in a Model E ultracentrifuge. The advantage of this type of cell is that sharp schlieren peaks are initially formed for the more or less polydisperse proteins under investigation (Woernley, Carruthers, Regent and Baumler, 1957). Sedimentation constants, $s_t$, were calculated from the slopes of log $x$ versus $t$ plots where $x$ is the distance in cm. of the schlieren peak from the axis of rotation at time $t$ in seconds. The $s_{20}$ values were obtained with the aid of Svedberg's reduction formula (Svedberg and Pedersen, 1940). The centrifugal force employed was 259,700 $\times$ g.

Viscosities were determined on an Ostwald-Fenske viscometer with a flow time for water of 245·5 sec. at 20° C. All samples for viscometry were subjected to centrifugation to remove sources of interference to capillary flow such as foreign particles. Mobilities were measured in the usual manner in a Perkin-Elmer Model 208 electrophoresis apparatus (Moore and White, 1948; Moore, Roberts, Costello and Schonberger, 1949).

### RESULTS

In Rudall's (1952) original studies on the fibrous and non-fibrous (structural) proteins of beef snout epidermis, maximum insolubility of the former at pH 5·5 and of the latter at pH 4·5 respectively was employed for isolation and purification procedures. The mobility of each of these proteins was determined in 0·2 ionic strength buffers (Miller and Golder, 1950) as a function of pH. Due to the insolubility of these proteins between pH 4 and 6, accurate measurement of the isolectric points was impossible. Fig. 1 and 2 show respectively the variation in mobility with pH of the fibrous and non-fibrous proteins of beef snout epidermis. In the electrophoresis of these protein preparations a single electrophoretic component was usually present; these patterns were quite sharp at the lower pH levels but became broader above pH 7·0. These observations indicate that the two proteins are fairly homogeneous.

Homogeneity is further indicated in the electrophoretic patterns of the alkali soluble protein (Fig. 3) and of the urea extractable protein (Fig. 4) of methylcholanthrene induced papillomas. These patterns were thus employed for the calculations of the mobility of the urea and alkali soluble proteins of normal and pathological epidermis.

The mobility of the urea extractable protein of the squamous cell carcinomas was somewhat greater than that of the protein of papillomas and of croton oil oil and methylcholanthrene treated epidermis (Table I). The average mobility of the alkali soluble protein (which precipitated maximally at pH 5·5 and hereafter denoted as the alkali soluble protein (Woernley, Carruthers, Regent and Baumler, 1957)) was nearly the same for the carcinomas, papillomas and treated epidermis. The average mobility of the urea and alkali soluble protein of human epidermis was greater than that of croton oil or methylcholanthrene treated mouse epidermis (Table I). Extraction of two samples of human basal cell carcinomas with 6M urea failed to yield any protein which precipitated at pH 5·5 although the one which flocculated at pH 4·5 was obtained in the usual manner.

FIG. 1.—pH-mobility curve of the fibrous protein of beef snout epidermis.

FIG. 2.—pH-mobility curve of the non-fibrous protein of beef snout epidermis.

TABLE I.—*Mobility of the Urea and Alkali Soluble Proteins of Epidermis*

| Tissue | Urea extractable protein mobility† cm.$^2$ volt$^{-1}$ sec.$^{-1}$ $\times 10^5$ | Alkali soluble protein mobility† cm.$^2$ volt$^{-1}$ sec.$^{-1}$ $\times 10^5$ |
|---|---|---|
| Croton oil treated epidermis | 2·3±0·3 (5)* | 3·4±0·3 (4) |
| Methylcholanthrene treated epidermis | 2·6±0·4 (4) | 3·0±0·3 (3) |
| Papillomas | 2·7±0·1 (2) | 3·1 (1) |
| Squamous-cell carcinomas | 3·0±0·3 (4) | 2·9±0·1 (3) |
| Human epidermis | 3·8±0·6 (5) | 4·2±0·1 (3) |
| Beef snout epidermis | 2·8±0·2 (5) | 3·4±0·2 (3) |

* Numbers of samples are given in parentheses. For the croton oil and methylcholanthrene treated groups 70 to 100 mice were used for each analysis and for the papillomas and carcinomas, about 300 and 20 mice respectively were employed.

† Electrophoresis was carried out in phosphate buffer—NaCl solution of pH 7·4 (Γ/2, 0·2) at 1·5° C.

The mobility of the proteins from beef snout epidermis was quite similar to that obtained for the proteins from mouse epidermis. The mobility of the urea and alkali soluble proteins of beef snout epidermis was not appreciably affected by pre-extraction of this tissue with a solution of ether and alcohol (equal volumes) or with ether alone. These experiments were carried out since the lipids of mouse and human epidermis had to be removed prior to the extraction of this tissue with 6M urea. This step was necessary since urea-lipid complexes, which formed creamy homogenates, prevented isolation of the various proteins.

The sedimentation constants, $s_t$, were determined at 3–5 different concentrations of the proteins in 0·05M borate buffer and in 0·05M borate in 6M urea. A plot of log $x$ versus $t$ where $x$ is the distance in cm. of the schlieren peak from the axis of rotation at time, $t$, in seconds, is shown in Fig. 5. The $s_{20}$ values were then obtained with the aid of Svedberg's reduction formula.

The average $s_{20}$ values and intrinsic viscosity $[N]$, of the urea and alkali-soluble proteins of mouse epidermis in various states are shown in Table II. There was no significant change in the average $s_{20}$ value of either protein in the epidermis undergoing carcinogenesis. The $s_{20}$ value of the urea extractable protein was nearly the same in borate buffer as in borate buffer-6M urea. However, the schlieren peaks of this protein were much sharper in borate-6M urea solution (Fig. 6) than they were in the borate buffer alone (Fig. 7). The $s_{20}$ constants of the alkali soluble proteins were larger than those of the urea extractable protein and they showed some decrease in borate-6M urea solution as compared to borate buffer alone.

There was no significant difference in the average intrinsic viscosity of the urea soluble protein of epidermis undergoing carcinogenesis (Table II) and the values

EXLANATION OF PLATE.

FIG. 3.—Electrophoretic pattern of the urea extactable protein from papillomas descending limb, in phosphate buffer-NaCl solution of pH 7·4 (Γ/2, 0·2) at 1·5° C.

FIG. 4.—Electrophoretic pattern of the alkali soluble protein from papillomas descending limb, in phosphate buffer-NaCl solution of pH 7·4 (Γ/2, 0·2) at 1·5° C.

FIG. 6.—Schlieren patterns for urea extractable protein of squamous-cell carcinomas ; 0·05M borate buffer in 6M urea ; concentration 0·66 g./100 ml. solution ; time interval between photographs, 8 min.

FIG. 7.—Schieren patterns for urea extractable protein of squamous-cell carcinomas ; 0·05M buffer ; concentration 1·08 g./100 ml. solution ; time interval between photographs, 8 min.

3

4

6

7

were nearly the same in borate buffer as in borate buffer-6M urea solution. The average intrinsic viscosity of the alkali soluble protein of the carcinomas in borate buffer was less than that of the treated epidermis. The average intrinsic viscosity of this protein in borate buffer-6M urea solution was nearly twice that found in the borate buffer alone for each tissue. The decrease in the intrinsic viscosity in order from croton oil treated and methylcholanthrene treated epidermis, papillomas

FIG. 5.—Typical log $x$ versus $t$ plot for urea extractable protein of croton oil treated epidermis.

TABLE II.—*Sedimentation Constant and Intrinsic Viscosity of the Urea and Alkali Soluble Proteins of Mouse Epidermis Undergoing Carcinogenesis*

| | Urea extractable protein in | | | | Alkali soluble protein in | | | |
|---|---|---|---|---|---|---|---|---|
| | 0·05M borate† | | 0·05M borate† in 6M urea | | 0·05M borate† | | 0·05M borate† in 6M urea | |
| Tissue | $s_{20}$ | [N] | $s_{20}$ | [N] | $s_{20}$ | [N] | $s_{20}$ | [N] |
| Croton oil treated epidermis | 1·8±0·2(3)* | 0·16±0·03(3) | 1·9±0·1(2) | 0·26±0·06(3) | 3·0±0·3(3) | 0·48±0·09(6) | 2·4±0·2(2) | 1·1±0·3(6) |
| Methyl-cholan-threne treated epidermis | 1·8±0·2(3) | 0·21±0·04(2) | 1·7±0·1(3) | 0·27±0·01(3) | 3·3±0·6(2) | 0·49±0·09(3) ) | 2·2±0·1(2) | 0·9±0·1(3) |
| Papillo-mas | 1·9±0·2(3) | 0·11±0·03(2) | 1·9 (1) | 0·33±0·07(2) | 3·5 (1) | 0·43±0·10(2) | — — | 0·8 (1) |
| Squamous-cell carci-nomas | 1·9±0·2(3) | 0·21±0·03(2) | 1·9±0·1(2) | 0·23±0·03(3) | 2·6±0·5(3) | 0·27±0·09(5) | 2·2±0·1(2) | 0·6±0·3(5) |

* Numbers of samples are given in parentheses. Number of mice used per analysis is the same as given in Table I.
† 0·05 moles $Na_2 B_4O_7 10H_2O$ dissolved in a liter of distilled water.

and carcinomas may be of some significance but more analysis would be necessary to prove this. The increase in intrinsic viscosity of the alkali soluble protein in borate buffer urea solution as compared to that in borate buffer alone is probably due to an increase in axial ratio or asymmetry of the protein molecule ((Putnam, 1953).

The $s_{20}$ and intrinsic viscosity of the urea and alkali soluble proteins of human and beef snout epidermis are shown in Table III. The $s_{20}$ and intrinsic viscosity values were of the same order of magnitude for the proteins of each type of epidermis and they were greater than for the same proteins of croton oil treated mouse epidermis. The decrease in $s_{20}$ of the alkali soluble proteins in borate-6M urea buffer as compared to the buffer alone was similar to that found for mouse epidermis.

TABLE III.—*Sedimentation Constant and Intrinsic Viscosity of the Urea and Alkali Soluble Proteins of Human and Beef Snout Epidermis*

| Type of epidermis | Urea extractable protein in | | | | Alkali soluble protein in | | | |
|---|---|---|---|---|---|---|---|---|
| | 0·05M borate† | | 0·05M borate† in 6M urea | | 0·05M borate† | | 0·05M borate† in 6M urea *f* | |
| | $s_{20}$ | [N] | $s_{20}$ | [N] | $s_{20}$ | [N] | $s_{20}$ | [N] |
| Human | 2·3±0·1(4)* | 0·27±0·07(4) | 1·6±0·1(3) | 0·48±0·11(3) | 4·2±0·1(3) | 0·73±0·13(4) | 2·4±0·1(3) | 0·91±0·19(4) |
| Beef snout | 2·1+0·3(4) | 0·28+0·07(5) | 1·8 (1) | 0·30+0·09(3) | 4·3 (1) | 0·54 (1) | 2·3 (1) | 0·67 (1) |

* Numbers of samples are given in parentheses.

† 0·05 moles $Na_2B_4O_7.10H_2O$ dissolved in a liter of distilled water.

The partial specific volume *V* of the urea extractable protein from croton oil treated epidermis was determined with borate buffer as the solvent, and assuming no solvation a value, $V = 0{\cdot}744$ c.c./g. was calculated from density measurements. In Table IV are listed viscosity increments, extrapolated to zero volume fraction (($\varphi = 0$), axial ratios, frictional coefficients, $s_0$ values and average molecular weights of the urea and alkali extractable proteins in 0·05M borate buffer. In the calculation for axial ratio frictional coefficient and molecular weight a prolate ellipsoid-shaped molecule was assumed (Golder, 1953 ; Simha, 1940).

From Table IV, it is seen that the urea extractable protein has a lower molecular weight than the protein extracted by alkali from the residue of the urea extracted epidermis. The latter in its original state is probably held together by stronger bonds than the former and the alkali soluble protein is probably more keratinous.

TABLE IV.—*Viscosity Increments, Axial Ratios, Frictional Coefficients, Sedimentation Constants and Molecular Weights of Proteins of Croton Oil Treated Mouse Epidermis in the Solvent,* 0·05M *Borate Buffer*

| Extractant | Viscosity increment ($\varphi = 0$) | Axial ratio | Frictional coefficient | $s_0$ Sveds | Molecular weight |
|---|---|---|---|---|---|
| 6M urea | 21·5 | 13·8 | 1·7 | 1·8 | 28,000 |
| 0·05N NaOH | 64·5 | 27·5 | 2·3 | 3·0 | 91,000 |

### DISCUSSION

The investigations of many workers have indicated that the adhesiveness of squamous cells is probably due to the nature of the proteins associated with intracellular bridges or tonofibrils (Giroud and Leblond, 1951; Mercer, 1949). Rudall's studies on the fibrous protein of epidermis indicated that this protein, a prekeratin, is a part of the tonofibril (Rudall, 1952). Hence, it appeared reasonable to assume that this protein might be of some importance in the carcinogenesis of epidermis. This is so because carcinomas of this tissue in man metastasize, a process requiring the freeing of cells or groups of cells from the main tumor mass. Actually, Coman (1947) has demonstrated that the force necessary to separate pairs of cells of squamous-cell carcinomas is much less than the force needed to pull apart pairs of normal epidermal cells.

The studies reported here on the physical-chemical properties of the urea and alkali soluble proteins of epidermis in various pathological states indicate possible trends only. The average mobility of the urea extractable protein of mouse epidermis undergoing carcinogenesis was highest for the carcinoma, and lowest for the croton oil and methylcholanthrene treated epidermis. Two samples of human basal-cell carcinomas were found to contain no measurable amount of urea extractable protein precipitable at pH 5·5, but the one which flocculated at pH 4·5 was isolated in the customary manner.

The $s_{20}$ constant and intrinsic viscosity of the urea extractable protein were of the same order of magnitude for the protein isolated from croton oil and methylcholanthrene treated epidermis, papillomas and carcinomas. The $s_{20}$ value of the alkali soluble protein was similar for these tissues, but there was a decrease in the average intrinsic viscosity for this protein in borate-6M urea buffer in the order croton oil and methylcholanthrene treated epidermis, papillomas and carcinomas. The schlieren peaks of the urea and alakli-soluble proteins were much sharper and the sedimentation constants were lower in the borate buffer-6M urea solutions than in the borate buffer alone. The latter difference is probably due to an increase in asymmetry of the protein molecules in the urea solutions.

### SUMMARY

Certain urea and alkali-soluble proteins of mouse epidermis undergoing carcinogenesis and of human and beef snout epidermis were investigated.

The average mobility of the urea extractable protein of croton oil and methylcholanthrene treated epidermis was lower than that of the papillomas and carcinomas. The average mobility of the alkali soluble protein of these tissues was not appreciably different.

The average sedimentation constant of the urea and alkali soluble proteins of croton oil and methylcholanthrene treated epidermis, papillomas and carcinomas were not appreciably different. The average intrinsic viscosity of the alkali soluble protein was greater for the croton oil and methylcholanthrene treated epidermis than for the carcinomas. The average intrinsic viscosity of the urea extractable protein of these tissues showed no appreciable difference.

The sedimentation constant was less and the schlieren peaks were sharper for the epidermal proteins in 6M urea borate buffer solution than in borate buffer alone. The former difference is probably due to the protein molecules being more asymmetrical in the urea solution than in the borate buffer alone.

### REFERENCES

Baumberger, J. P., Suntzeff, V. and Cowdry, E. V.—(1942) *J. nat. Cancer Inst.*, **2**, 413.

Carruthers, C.—(1950) *Cancer Res.*, **10**, 255.

*Idem*, Woernley, D. L., Baumler, A. and Kress, B.—(1955) *J. invest. Derm.*, **25**, 89.

*Idem*, WOERNLEY, D. L., BAUMLER, A. AND SHORTS, H.—(1955) *J. Soc. cosmet. Chem.*, **6**, 324.
*Idem*, WOERNLEY, D. AND HITTLEMAN, J.—(1957) *J. invest. Derm.*, **29**, 39.
COMAN, D. R.—(1947) *Science*, **105**, 347.
GIROUD, A. AND LEBLOND, C. P.—(1951) *Ann. N.Y. Acad. Sci.*, **53**, 613.
GOLDER, R. H.—(1953) *J. Amer. chem. Soc.*, **75**, 1739.
MERCER, E. H.—(1949) *Biochem. Biophys. Acta*, **3**, 161.
MILLER, G. L. AND GOLDER, R. H.—(1950) *Arch. Biochem.*, **29**, 420.
MOORE, D. H. AND WHITE, J. U.—(1948) *Rev. sci. Instrum.*, **19**, 700.
*Idem*, ROBERTS, J. B., COSTELLO, M. AND SCHONBERGER, T. W.—(1949) *J. biol. Chem.*, **180**, 1147.
PUTNAM, W. F.—(1953) *in* Neurath, H. and Bailey, K. ' The Proteins '. New York (Academic Press), 1B, 847.
ROE, F. J. C.—(1956) *Brit. J. Cancer*, **10**, 72.
RUDALL, K. M.—(1952) *Advanc. Protein Chem.*, **7**, 253.
SIMHA, R.—(1940) *J. phys. Chem.*, **44**, 25.
SVEDBERG, T. AND PEDERSEN, K. O.—(1940) ' The Ultracentrifuge '. Oxford (Clarendon Press), p. 35.
WOERNLEY, D. L., CARRUTHERS, C., REGENT, L. AND BAUMLER, A.—(1957) *Arch. Biochem.*, **66**, 167.